# Colon Cancer Biomarkers: Implications for Personalized Medicine

**DOI:** 10.3390/jpm10040167

**Published:** 2020-10-13

**Authors:** Kenneth P.H. Pritzker

**Affiliations:** Department of Laboratory Medicine and Pathobiology, Temerty Faculty of Medicine, University of Toronto, Toronto, ON M5S 1A8, Canada; kenpritzker@gmail.com

**Keywords:** colon cancer biomarkers, predictive biomarkers, prognostic biomarkers, personalized medicine, colon cancer, colon cancer heterogeneity

## Abstract

The heterogeneity of colon cancers and their reactions presents both a challenge and promise for personalized medicine. The challenge is to develop effective biologically personalized therapeutics guided by predictive and prognostic biomarkers. Presently, there are several classes of candidate biomarkers, including genomic probes, inhibitory RNAs, assays for immunity dysfunction and, not to be forgotten, specific histopathologic and histochemical features. To develop effective therapeutics, candidate biomarkers must be qualified and validated in comparable independent cohorts, no small undertaking. This process and subsequent deployment in clinical practice involves not only the strong association of the biomarker with the treatment but also careful attention to the prosaic aspects of representative tumor site selection, obtaining a fully adequate sample which is preserved and prepared to optimize high quality analysis. In the future, the clinical utility of biomarker analytical results will benefit from associated clinical and basic science data with the assistance of artificial intelligence techniques. By application of an individualized, selected suite of biomarkers, comprehensively interpreted, individualized, more effective and less toxic therapy for colon cancer will be enabled, thereby fulfilling the promise of personalized medicine.

As noted almost two decades ago and still applicable now, developing clinically useful biomarkers for cancer is much easier said than done [1]. To become clinically useful, beyond association with a particular facet of diagnosis or therapy, cancer biomarkers require clarity of intended use, accurate, easily reproducible quantitative assays, as well as extensive independent validation [2,3,4].

It is widely perceived and has been selectively demonstrated [5,6] that reduction in colorectal cancer mortality and morbidity will be achieved by deployment of personalized medicine, more precisely, biologically personalized therapeutics [7]. This goal requires qualified biomarkers to select potentially useful therapy for individuals and to assess their response to treatment. As the prime focus of cancer biologists in recent years has been genomics and immunobiology, most biomarker development has been set towards molecular markers predictive for efficacy of specific targeted drug classes and towards markers prognostic for tumor–host responses.

Recently, Boussios et al. reviewed prominent biomarker classes used, or candidates for clinical use, for biologically personalized therapeutics in colorectal cancer [8]. This review focused on predictive molecular biomarkers with emphasis on mismatch DNA repair genes, mutation markers for genes in the Mitogen-Activated Protein Kinase (MAPK) pathway such as KRAS, NRAS and BRAF as well as miRNAs. As well, this review discussed some prognostic markers including those associated with inflammation as found variously in blood or stool.

Stepping back from the details of clinical studies carefully and extensively assembled, the data illuminated in this review sharply revealed several insights.

## 1. Cancers at Different Anatomical Sites Display Different Predictive Biomarkers but the Significance of Molecular Subtyping in Improving Colon Cancer Survival Remains to Be Demonstrated

While it has been long known that right side colorectal cancers are more aggressive than on the left [9], recent studies have shown that the pattern of genomic oncogenic changes within the tumors differs according to anatomical location [10,11]. These observations demonstrate molecular heterogeneity of colon cancers at the same stage and the need to have a comprehensive study of such changes in each patient to establish whether targeted therapies might be successful [12,13]. Lacking in most of these studies is comparison of outcomes with the standard of care assessment for tumor cancer aggressiveness, TNM (Tumor, Node, Metastasis) staging, histologic type and grade [13]. This gap needs to be rectified before the contextual significance of genomic variation can be understood well.

## 2. Limited Efficacy to Date of Molecular Target-Based Therapies in Colorectal Cancer

As Boussios et al. show, only a minority of colorectal patients exhibit response to targeted therapy and most of these responses are limited to a few months’ duration [8]. This indicates that pivotal molecular targets and their drugs remain to be found for colorectal cancer.

## 3. Lack of Success of “Tumor Agnostic Molecular Therapies” for Colorectal Cancer

Site-agnostic molecular markers are being avidly pursued for targeted drugs [14]. However, the differential responses of cancers at different anatomical sites and particularly the modest responses of colon cancers against drugs which are more effective for other cancers, reinforces that colon cancers are heterogenous in their pathophysiology. This suggests that before a panacea cancer therapeutic or diagnostic strategy can be considered, much more knowledge is required.

## 4. Differential Selection Criteria Should Apply for Screening and Prognostic Markers Compared to Predictive Biomarkers

The clinical utility of a biomarker that is predictive for a response by a molecularly targeted drug must be assessed by the alternatives of not providing the drug at all or providing the drug without biomarker assessment. These choices are clear. To not provide the drug deprives the patient of potential benefit even if the benefit may apply to only a small minority of patients and has a short duration. To provide the drug without predictive biomarker testing exposes the majority of patients to toxic therapy without benefit and may compromise their capacity to respond to other agents. With regard to screening biomarkers, the adverse social and economic costs of false positives on a population basis must be weighed against the values of finding incremental asymptomatic cases beyond current screening recommendations [15,16]. For prognostic markers, the key selection factor is not whether the marker is prognostic but how much more prognostic the marker might be compared to standard of care diagnostics. The cost/benefit decision to deploy a prognostic marker needs to be governed by clinical utility, namely what clinical decisions might be made with the biomarker result.

## 5. Opportunity to Combine Colorectal Biomarkers from Different Classes as Personalized Medicine “Prognostic Signatures”

Biomarker signatures are commonly explored as a means of increasing biomarker sensitivity. However, as Boussios et al. demonstrated [8], the usual practice is to develop markers within a single class such as gene mutations, or miRNA expression. While convenient for analysis within a single discipline or laboratory, such signatures are indicative of only one or a few biologic processes and therefore have limited clinical utility to be predictive or prognostic of the complex cellular environments present in cancer. The opportunity exists to explore biomarker signatures between classes of markers with a marker from each class selected for its capability to best assess different relevant phenomena such as cell replication, tissue invasion and metastatic potential. Advances in artificial intelligence which have already been applied in metastatic colorectal cancer to correlate KRAS, NRAS and BRAF with clinical pathologic parameters [17] to colonoscopy assessments [18], and to colon cancer image analysis [19,20], are likely to accelerate success of the opportunity to develop biomarker signatures based on mixed classes of markers, such as proteomics and morphology [21]. To enable this goal, artificial intelligence would be used together with a large dataset obtained by analysis of samples from a patient and compared to a reference dataset compiled from previous studies. Together, the tool becomes a computer-assisted diagnostic for personalized medicine. It is possible that the diagnostic may be so powerful that it can be deployed as a predictive marker for colon cancer therapy on the basis of retrospective studies, just as KRAS was in 2008–2009 [22,23]. Nonetheless, prospective studies are the usual requirement for test validation and should be undertaken, if only to provide additional diagnostic insights.

## 6. Improving Predictive Biomarkers by Improving Sample Accession Techniques

Critical to the success of biomarkers is the care taken to obtain the best quality samples for each analytical technology. For example, for radiologic and colonoscopy observations, real-time recording of regions of interest supplemented by artificial intelligence promises to supply information of higher quality than current standard of care. Bussios et al. included in their review a discussion of markers for cancer aggressiveness and immunologic reaction to cancer using blood, “liquid biopsy” and stool [8]. At best, samples from these sources usually require techniques much more sensitive than for those obtained directly from the tumor; in real practice the samples obtained by these methods may contain ambient substances that degrade the analyte or interfere with analysis. Moreover, samples from blood or stool may not be fully representative of the analyte status within the tumor. These techniques are used primarily because the prospect of obtaining adequate material from biopsies, particularly metastases, is considered fraught with difficulties of adequate sampling and biopsy procedure complications [24]. However, compared to liquid biopsy, and despite the great need for better conventional biopsies, to date, there has been relatively little investigative attention to the improvement of needle biopsy techniques with regard to yield and sample quality.

## 7. Predictive Markers Need to Be Performed Immediately before Therapy Selection

As an individual’s colorectal cancer advances, the cancer’s cell biology changes as a result of mutations but, even more relevant, cancer cell-cell interaction, clonal selection by chemotherapy, radiotherapy, targeted therapy and/or immunotherapy, as well as inflammatory/immunological responses. This implies that an individual’s cancer’s biology should be evaluated for predictive markers immediately preceding therapy selection. In future diagnostic assessments this may include predictive and prognostic markers for the functional status of colon cancer stem cells [25,26].

Boussios et al.’s review demonstrated that predictive and prognostic biomarkers for colon cancer has become a vast and, at present, confusing field [8]. This relates directly to the biological heterogeneity both of the cancer and the host individual. The selection of appropriate personalized therapy will require appropriate specific markers, with the large amount of data likely guided at least in part by artificial intelligence techniques. As noted above, careful biomarker selection and selection of the most appropriate representative tumor sample, together with optimized sampling, as well as high-quality sample preparation and analysis, are likely prerequisites for successful biologically personalized therapeutics for colon cancer.
